# Hypertension and Risk of Post-Operative Cognitive Dysfunction (POCD): A Systematic Review and Meta-Analysis

**DOI:** 10.2174/1745017901713010027

**Published:** 2017-05-18

**Authors:** I. Feinkohl, G. Winterer, T. Pischon

**Affiliations:** 1 Molecular Epidemiology Research Group, Max-Delbrueck-Center for Molecular Medicine in the Helmholtz Association (MDC), Berlin, Germany; 2 Charité – Universitaetsmedizin Berlin, Berlin, Germany; 3 MDC/BIH Biobank, Max-Delbrueck-Center for Molecular Medicine in the Helmholtz Association (MDC), and Berlin Institute of Health (BIH), Berlin, Germany

**Keywords:** Cognitive epidemiology, Blood pressure, Hypertension, Post-operative cognitive dysfunction, POCD, Meta-Analysis

## Abstract

**Background:**

Post-operative cognitive dysfunction (POCD) occurs frequently after major surgery. Hypertension is well-established as a risk factor for age-related cognitive impairment, but it is unclear whether or not it also increases the risk of POCD.

**Objective:**

To evaluate the role of hypertension in POCD risk in a systematic review and meta-analysis.

**Method:**

PubMed, Ovid SP and the Cochrane Database of Systematic Reviews were searched for longitudinal studies of adults undergoing surgery with reporting of hypertension, blood pressure and/or anti-hypertensive treatment associations with POCD as relative risks or odds ratios. Fixed-effects meta-analyses were performed using Review Manager (version 5.3).

**Results:**

Twenty-four studies on 4317 patients (mean age 63 years) were included. None of the studies had set out to assess hypertension as a risk factor for POCD. Hypertension was used as a categorical predictor throughout and only 2 studies adjusted for potential confounders. Across all 24 studies, hypertension was not significantly associated with POCD risk (RR 1.01; 95% CI 0.93, 1.09; *p*=0.82), though among 8 studies with >75% males, we found hypertension associations with a 27% increased risk of POCD (RR 1.27, 95% CI 1.07, 1.49; *p*=0.005).

**Conclusion:**

Our findings do not support the hypothesis that hypertension is a risk factor for POCD. However, since none of the studies included in our analysis were hypothesis-driven and most did not adjust for potential confounders, further systematic investigations are needed to evaluate the role of hypertension in the epidemiology of POCD.

## INTRODUCTION

Post-operative cognitive dysfunction (POCD) occurs frequently after major surgery [1]. It is broadly defined as an impairment of a patient’s cognitive functioning relative to their pre-surgery cognitive status [2]. POCD is considered transient [2] but may remain detectable for months and years after surgery [3]. In patients with persistent POCD, it is known to negatively impact on everyday life tasks [4], quality of life [5], subjective memory performance [6], emotional symptoms [7], and may predict more severe health consequences such as dementia and premature mortality [2, 8, 9]. Both the prevalence of hypertension and the likelihood of major surgery increase with advanced age [10-13]. Indeed, hypertension is extremely common across the Western world, with approximately 30% of adults affected in the US [14], and it is a well-established risk factor for cognitive impairment in older ages [15]. Yet, it is entirely unclear, whether or not patients with hypertension are also at increased risk of POCD. A role of hypertension as a risk factor for POCD is plausible on the basis that it increases the risk of post-operative delirium (POD) [16], which itself is strongly linked to POCD. Further, as part of the metabolic syndrome, hypertension often occurs in people with diabetes or obesity [17], which both have recently been identified as potential risk factors for POCD [18, 19], and it is common in surgical patients [6, 20]. Hypertension is potentially modifiable by using relatively cost-effective measures, including modification of diet and lifestyle or drug-treatment [21]. Therefore, any association of hypertension with risk of POCD would have far-reaching implications for risk assessment in surgical patients and – potentially – for prevention of POCD. The objective of our study was therefore to conduct a systematic review and meta-analysis on epidemiological studies of hypertension, blood pressure and anti-hypertensive treatment prior to surgery and risk of POCD.

## MATERIALS AND METHODS

### Systematic Search Strategy

The PubMed, Ovid SP and Cochrane Database of Systematic Reviews were searched from their respective inception to 25^th^ April 2016. Titles and abstracts were searched for the following terms: (((blood pressure OR systolic OR diastolic OR antihypertens* OR hypertens*))) AND ((post-operative cognit* OR postoperative cognit* OR POCD) OR ((surgery OR operation) AND (cognit OR intelligence OR MMSE OR Mini Mental OR dementia OR Alzheim* OR mild cognitive impairment OR MCI))). All titles and abstracts of articles that remained following removal of duplicates were screened against inclusion criteria by one investigator (IF). If they were deemed to potentially match inclusion criteria or if they appeared to have data on both hypertension and POCD (*e.g.*, adjusted analyses of POCD for hypertension), full texts were accessed. Reference lists of any review articles identified in the search and of included studies were screened for further original articles that also entered the full text review stage. The search adhered to MOOSE [22] and PRISMA [23] guidelines, and was registered on the PROSPERO database (Registration No. CRD42016038236).

### Study Selection

We included studies that fulfilled all of the following criteria: i) prospective study of any design ii) sample of human adults (≥18 years old) undergoing surgery iii) full text in English language iv) ascertainment of blood pressure, hypertension and/or antihypertensive treatment prior to surgery v) reporting of these exposure variables with risk of POCD as relative risks (RR) or odds ratios (both taken as RR for the purpose of the present analysis, as odds ratios and RR are close to identical in assessments of rare outcomes [24]) or in a form that allowed calculation of RR.

Any type of surgery, any definition of POCD and any length of follow-up qualified for inclusion. Use of the term ‘POCD’ was not required. Studies on post-operative delirium, on hypotension or on blood pressure during surgery or in the post-operative period were not considered. Corresponding authors were contacted for any essential unreported information unless previous contact had been unsuccessful. That way, unpublished data were obtained for one article [25]. If an article lacking essential unreported information was suspected of duplicate reporting of another article that provided sufficient detail, the latter was selected for inclusion. 

### Data Extraction

For each article, RR statistics on the respective longest follow-up period were extracted. Preference was given to fully adjusted multivariate models unless no adjustment was made. Data were tabulated for separate meta-analysis of each predictor as appropriate.

For one study which compared patients who had “improved” versus “not improved” on cognitive tests, “not improved” was used to represent POCD for the purpose of the present analysis [26]. For another that assessed three levels of cognitive change, “severe deterioration” was considered as POCD and contrasted with “no deterioration” and “mild deterioration” [27]. Another study compared various levels of cognitive impairment and we equated “major decline” with POCD [28]. Finally, one study assessed improvement in cognitive function after 1 year in a sample of patients who all were classified to suffer from POCD at 6-week follow-up [6]. “No improvement” was taken to represent POCD for that study. We included one study in which baseline cognitive assessment was performed *after* rather than prior to surgery in a small proportion (18%) of patients [28].

For two studies, the originally reported upper limits of the 95% confidence intervals of their estimates were implausible, and for the purpose of the present analysis were calculated on the basis of the respective lower limit [29, 30].

### Data Synthesis

Extracted statistical data were entered into Review Manager (version 5.3; the Cochrane Collaboration) to calculate summary estimates in inverse variance fixed-effects models. Statistical heterogeneity was indexed by I^2^ and publication bias was evaluated through visual inspection of funnel plots and Egger’s regression analysis [31]. Multiple fixed-effects meta-regression analyses explored differences between subgroups of studies. Specifically, studies were compared according to follow-up period (≤1 month versus >1 month), sample size (≤100 versus >100), mean sample age (≤65 years versus >65 years), surgery type (cardiac; non-cardiac; mixed surgery type) and sex (≤75% males versus >75% males). All cut-points for subgroup analyses were selected a priori to obtain around equally sized groups of studies without any pre-specified hypotheses. For example, for “sex”, the cut-point was selected on the basis that studies of POCD are often skewed toward inclusion of a greater proportion of males, because many studies focus on cardiac surgery which is more common in males than females [32]. We therefore expected a cut-point at 75% males to result in two around equally sized groups of studies. Meta-regression was performed using SAS Enterprise Guide (version 4.3).

### Quality Assessment

Both cohort and trial studies were scored by one investigator (IF) on the 22-item STROBE checklist of cohort studies [33], as all analyses on hypertension and POCD were observational in essence. No exclusion was applied based on STROBE scores.

## RESULTS

### Study Characteristics

**Fig. (1):**
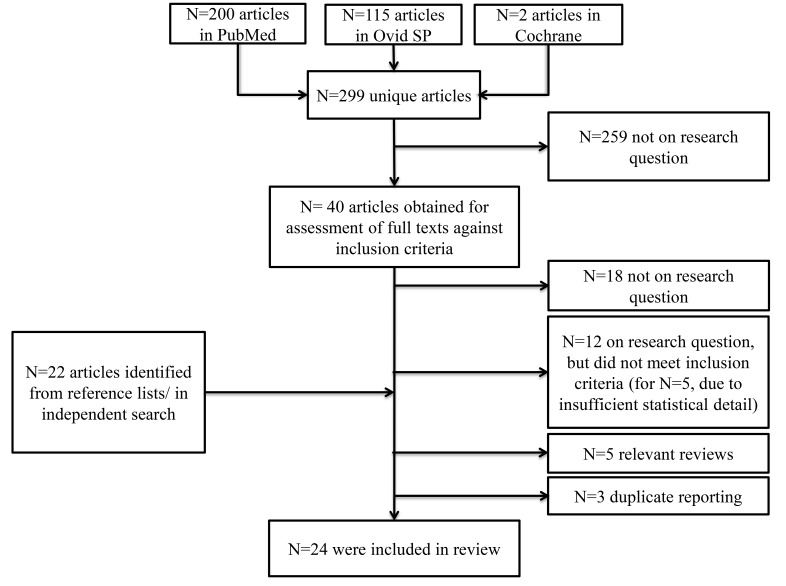
Flow chart of systematic search.

The search yielded N=200 articles in PubMed, N=115 articles in Ovid SP and N=2 articles in the Cochrane Database. Following removal of duplicates, N=299 articles remained for screening (Fig. **1**).

At this stage, 259 articles were excluded most commonly due to focusing on unrelated research topics including delirium, intra- or post-operative blood pressure or animal studies, or due to reporting of cognitive function as an exclusion criterion. Thus, full texts of 40 articles were accessed. Six articles qualified for inclusion of which 3 were excluded [34-36] due to suspected duplicate reporting of other articles with more complete reporting [9, 30, 37]. Twelve articles that addressed the research question were excluded due to failing to formally meet inclusion criteria but were considered qualitatively in sensitivity analyses. One article on cognitive symptoms following shunt surgery in hydrocephalus was excluded despite formally meeting inclusion criteria due to the neurosurgical nature of the surgery [38]. Screening of reference lists and an independent search identified 22 further relevant studies of which 21 met inclusion criteria. Overall, 24 articles were included [6, 9, 25-30, 37, 39-53].

Publication dates spanned 1980 to 2015 and studies originated in Europe, North America, Asia and Australia (Table **1**). Analysis samples included a total of 4317 patients. Sample characteristics and study designs were heterogeneous. Mean age (where reported) ranged from 42 to 75 years (mean 63 ± 7 years). Samples included between 29% and 81% males (where reported) though 19 of 24 studies included more males than females. Patients were followed up for between 1 day and 5 years after surgery (median 36 days, interquartile range 7 to 90 days). Procedures included cardiac (N=16), non-cardiac (N=7) and mixed (N=1) types of surgery.

All articles were on hypertension rather than systolic or diastolic blood pressures as linear measures. In the majority of studies (n=21), we found no information on how hypertension was defined or assessed. In 1 study, it was defined as systolic blood pressure >140 mmHg or use of anti-hypertensive treatment [41], and in another as use of anti-hypertensive medication though it is unclear whether or not blood pressure readings were additionally considered [49]. One study determined hypertension from self-report which was verified using medical records [25]. Among all 24 articles, only 2 explicitly referred to arterial hypertension [6, 47] but we assume that all evaluated arterial rather than other forms of hypertension. Where reported, hypertension was present in between 14% of patients in a study of relatively young Asian patients (mean age 42 years [50]) and 99% of patients in an older German sample (mean age 69 years [6]). The Mini Mental State Examination (or national equivalent) was administered in 5 studies [28, 29, 50, 52, 53]; all other studies used more detailed neuropsychological tests. Definition of POCD varied. In 6 studies, it was based on cognitive change relative to a non-surgical control group. POCD occurred in between 9% [27] and 75% [52] of patients. Statistical analyses of hypertension associations with POCD risk were adjusted for sociodemographic and clinical covariates in only 2 of the 24 studies [27, 37]; all of the remaining analyses reported unadjusted RR statistics or descriptive data that allowed calculation of univariate RR.

### Findings of Included Studies and Meta-Analysis: Hypertension

**Fig. (2):**
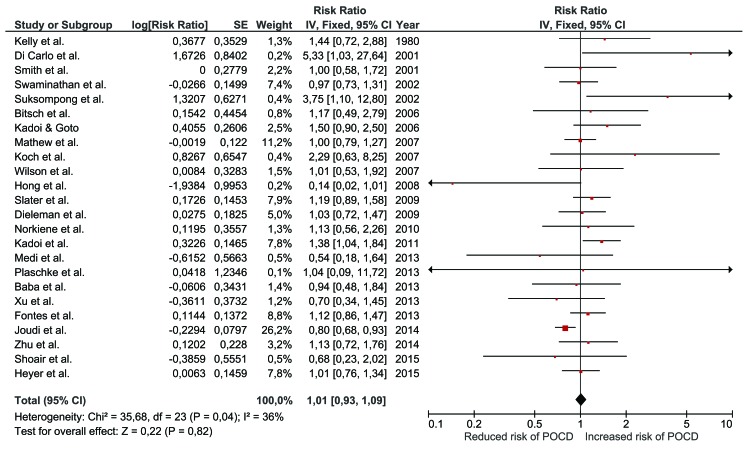
Forest plot of meta-analysis on hypertension and POCD risk.

**Fig. (3):**
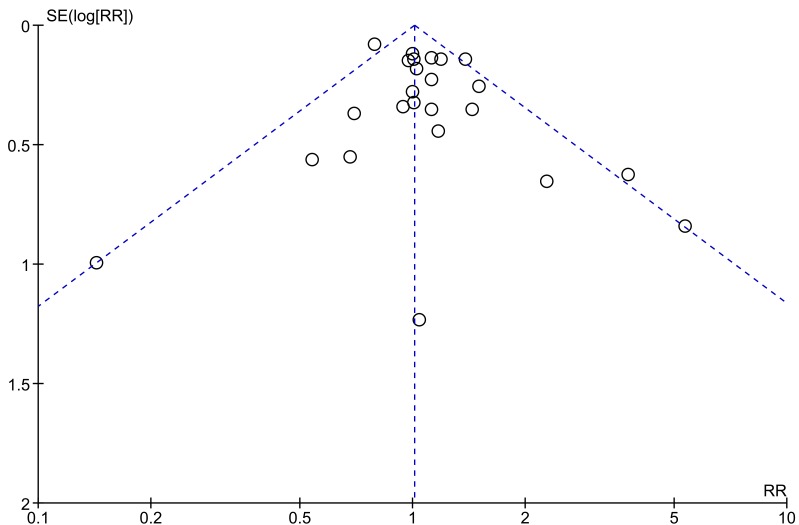
Funnel plot of meta-analysis on hypertension and POCD risk.

All included studies were on hypertension and so were entered into a single meta-analysis (Fig. **2**). Overall, there was no association between hypertension and risk of POCD (RR 1.01; 95% CI 0.93, 1.09; *p*=0.82). This risk estimate represents a largely unadjusted relationship of hypertension with POCD as only 2 studies applied statistical adjustment [27, 37]. The finding was similar when the analysis was repeated using a random-effects model (RR 1.06; 95% CI 0.94, 1.19; *p*=0.34). Statistical heterogeneity between studies was low to moderate (chi^2^ (23)=35.68; *p*=0.04; I^2^=36%) with no evidence of publication bias (Fig. **3**; Egger’s regression analysis, *p*=0.129).

### Subgroup Analyses and Meta-Regression: Hypertension

**Fig. (4):**
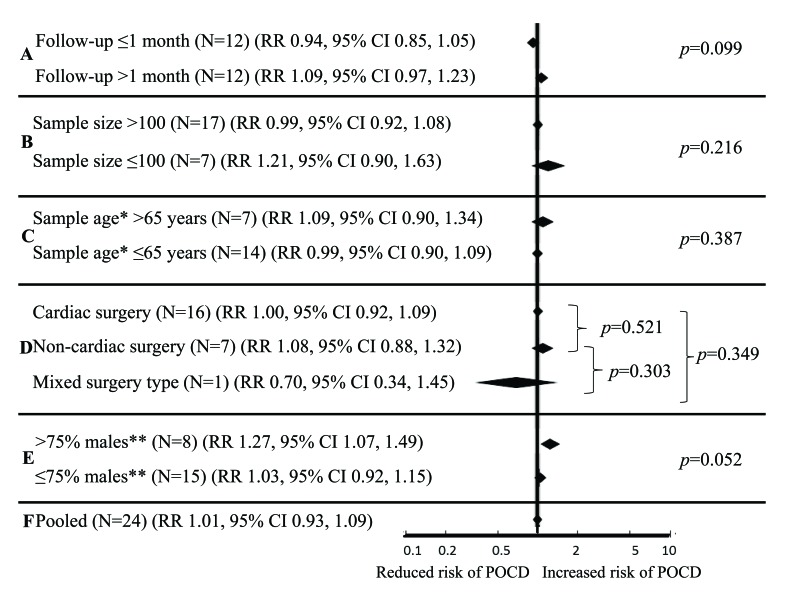
Analyses of subgroups of studies according to **A**) follow-up, **B**) sample size, **C**) mean sample age, **D**) surgery type and **E**) proportion of males, and **F**) overall pooled effects.*data missing for N=3 studies.**data missing for N=1 study.

Results of subgroup analyses are summarized in Fig. (**4**). Associations of hypertension with risk of POCD were statistically non-significant in all subgroups of studies based on follow-up period (≤1 month, RR 0.94, 95% CI 0.85, 1.05; >1 month RR 1.09, 95% CI 0.97, 1.23; meta-regression *p*=0.099), sample size (≤100, RR 1.21, 95% CI 0.90, 1.63; >100, RR 0.99, 95% CI 0.92, 1.08; meta-regression *p*=0.216), mean sample age (≤65 years, RR 0.99, 95% CI 0.90, 1.09; >65 years, RR 1.09, 95% CI 0.90, 1.34; meta-regression *p*=0.387) and type of surgery (cardiac, RR 1.00, 95% CI 0.92, 1.09; non-cardiac RR 1.08, 95% CI 0.88, 1.32; mixed RR 0.70, 95% CI 0.34, 1.45; meta-regression *p*=0.303 to *p*=0.521). However, when analyses were restricted to 8 studies with >75% males, hypertension was overall associated with a 27% increased risk of POCD (RR 1.27, 95% CI 1.07, 1.49; *p*=0.005). Of these, a single study applied statistical adjustment for sociodemographic and clinical covariates [37] so that the pooled estimate is largely unadjusted. Studies on ≤75% males revealed no association of hypertension with POCD (RR 1.03, 0.92, 1.15). The difference in risk estimates between these two groups of studies (≤75% males versus >75% males) approached statistical significance (meta-regression *p*=0.052; Fig. **4**).

### Qualitative Summary of Relevant Excluded Studies

Several studies strictly failed to meet inclusion criteria but may supplement our analyses. Five studies were on hypertension and POCD but were excluded due to lack of statistical detail [8, 54-57]. Here, hypertension associations with POCD were described in narrative form only [54-57], or descriptive data were insufficient to calculate RR [8]. Of these studies, all except one [56] found no association of hypertension with POCD. Other studies that failed to meet inclusion criteria on the basis of study design revealed more mixed evidence. In one imaging study, hypertension was unrelated to changes in the P300 component reflective of cognitive processing across surgery [58]. An analysis of hospital records showed interaction effects of hypertension with exposure to surgery in prediction of dementia diagnosis [59]; another reported no such evidence [60]. Three studies on continuous cognitive change reported null or marginal findings [61, 62] or detrimental effects [63] of hypertension. Finally, one study that did not differentiate between POCD and POD reported a *lower* risk of these outcomes in patients with hypertension [64].

## DISCUSSION

Here, we set out to combine the current epidemiological evidence on associations of pre-surgery hypertension, blood pressure and anti-hypertensive treatment with risk of post-operative cognitive dysfunction (POCD). All included articles were on hypertension and overall, we found little evidence of an association with POCD. However, all studies were of exploratory nature, and only 2 studies adjusted for potential confounders and, therefore, our meta-analysis does not rule out a (potentially causal) relationship. In subgroup analyses, we also found that among studies with proportion of males >75%, hypertension statistically significantly increased the risk of POCD by 27%. The finding warrants confirmation but may support hypertension as a contributing factor to POCD risk in a sub-set of patients.

There is a great deal of interest in hypertension as a cognitive risk factor due to high prevalence in the general [14], older [11], and in surgical populations [6, 20], and because it is modifiable. Anti-hypertensive treatment has been linked to a reduced risk of age-related cognitive impairment [65], though a Cochrane review of randomized controlled trials – which help shed light on the issue of causality – found that the overall evidence on anti-hypertensive treatment and risk of cognitive impairment was inconclusive [66].

A number of candidate contributors to reports of blood pressure links with cognitive risk [15, 67] have been identified and complex interplays among a range, or all, are likely. Fifty percent of patients with hypertension are affected by insulin resistance which impairs cognitive function directly for instance through alterations in cerebral blood flow, as well as indirectly through associated inflammatory response [68]. Disease of the cerebral vasculature as the basis of the increased risk of cognitive impairment seen in people with hypertension [69] finds support in reports of a reduced risk of cerebral infarction following improved blood pressure control [70]. In line with a now well-established vascular component of Alzheimer’s disease [68, 71], hypertension is further associated with deposition of the beta amyloid peptide [72]. Recently, low beta amyloid in cerebrospinal fluid (indicative of pre-clinical early stages of Alzheimer’s disease neuropathology) has also been linked to the development of POCD [73]. On the basis of that type of evidence, the present null finding across all included studies is surprising, but may be due to a number of factors. Statistical power was limited by high prevalence of hypertension in some studies (*e.g.*, 99% [6]). We further suspect ascertainment bias. Patients with poor health may not have undergone as detailed blood pressure assessment as healthier patients so that hypertension remained undetected. At the same time, these patients may have been prone to POCD. Definition of hypertension was rarely specified, and as is common in the research literature [41, 49, 74] likely often included the criterion “use of anti-hypertensive treatment”. This is despite uncertainty on its relationship with risk of age-related cognitive impairment per se [65, 75] and a lack of knowledge of its relationship with POCD. Hypertension could also have been well-controlled for years in patients on anti-hypertensive treatment. Finally, normotensive patients may have suffered from white-coat [76] and anxiety-induced hypertension due to scheduled surgery [77]. Overall, any actual underlying links of blood pressure with POCD risk may have been eliminated by such “dilution” of “hypertension” groups.

None of these explanations could reasonably account for reports of associations of hypertension with age-related cognitive impairment [15, 67, 78] and POD [16], however. All studies of hypertension would be equally affected. We therefore have to consider the possibility that our finding is not due to bias but reflects some difference of (potentially sex-specific) hypertension links with POCD versus other forms of impairment. This would be consistent for instance with associations of hypertension with risk of stroke [69] but mixed results for post-operative stroke in particular [79, 80].

We are unable to determine this on the basis of our results. From a clinical perspective, our findings indicate that hypertension at the time of presenting for surgery provides little information on the cognitive risk of a patient. However, the exploratory nature of the studies included here has to be considered. None set out to assess hypertension and risk of POCD. Only 2 of 24 included studies applied statistical adjustment, and these 2 built large statistical models without any pre-specified hypotheses. Thus, our finding should be seen as preliminary pending evaluation in further epidemiological studies targeted at the research question. With sex as a potential risk modifier, male and female samples would ideally be investigated separately. Blood pressure readings and use of anti-hypertensive medication (leading to normalization of blood pressure) should also be considered separately and studies should attempt to capture samples that include hypertensive and hypertension-free patients at equal proportion. The role of cognitive reserve, which predicts both late-life hypertension [81] and POCD [82], as well as potential interaction effects of hypertension with intraoperative blood pressure control warrant evaluation. Finally, frailty, which is related to blood pressure control [83], may be an important concept to recognize in cognitive epidemiology [84] including that of POCD.

A number of limitations must be considered. POCD definition was heterogeneous across studies and definitions of hypertension were generally lacked. Thus, we are unable to tease out the influence of blood pressure versus anti-hypertensive treatment on POCD risk. Statistical analyses in the primary studies were rarely adjusted for potential confounders. For sex in particular, the present analysis indicated that hypertension associations with POCD may be limited to samples that include a large proportion of males. Therefore, an influence of confounding by factors such as sex on our pooled estimates is likely. We performed several statistical tests in stratified analyses, which introduced risk of type I error; thus, these subgroup results are to be interpreted cautiously.

We conclude that current research studies do not support the hypothesis that hypertension is a risk factor for POCD; however, these studies had not set out to investigate the risk associated with hypertension and rarely considered potential confounding factors in their analyses. Adequately designed studies are urgently needed to elucidate the definitive role of hypertension in the epidemiology of POCD.

## Figures and Tables

**Table 1:** Summary of included studies.

Author, year, location	Total N enrolled in study	N completed follow-up	Male	Type of surgery, anesthesia	Mean age ± SD or median (IQ)	Follow-up	Cognitive measurement	Definition/ incidence of POCD	Hypertension exposure	Adjustment variables	Original reporting of exposure association with POCD as descriptive data and/or RR (95% CI)	STROBE score
Kelly *et al.* (1980) USA	41	35	66%	Carotid end-arterectomy General anesthesia	62 ± 8	4 to 8 weeks	12 neuro-psychological tests “Improved” defined as improvement on ≥1 test with no deterioration on any other, or improvement on ≥2 tests with deterioration on ≤1 other test	“Not improved” used as POCD and compared with “improved” in present analysis. POCD in n=19/35 (54.3%).	Hypertension not defined. Hypertension in n=21/35 (60.0%) of patients.	None	8/16 (50.0%) “improved” had hypertension. 13/19 (68.4%) “not improved” had hypertension.	13/22
Smith *et al.* (2000) North America	381	319	81%^b^	CABG General anesthesia	40% >65 years^b^	1 month	9 neuro-psychological tests	POCD defined as decline of ≥20% on ≥2 of tests. POCD in n=69/319 (21.6%).	Hypertension not defined. Hypertension in n=227/381 (59.6%) of patients^b^	None	RR 1.0 (0.58, 1.72)	19/22
Di Carlo *et al.* (2001) Italy	123	110	71%	CABG or intra-cardiac surgery General anesthesia	64 ± 9	6 months	4 neuro-psychological tests; MMSE. Rating by 2 neuropsychologists as “unchanged/improved”, “mild deterioration”, “severe deterioration”	“Severe deterioration” used as POCD and compared with “unchanged” in present analysis. POCD in n=10 /110 (9.1%).	Hypertension not defined. Hypertension in n=60/110 (54.5%) of patients.	Education, partial pressure of carbon dioxide (only significant predictors retained in final model along with hypertension)	RR 5.33 (1.03, 27.64)	19/22
Suksompong *et al.* (2002) Thailand	110	110	76%	CABG General anesthesia	62 ± 8	3 to 5 days	Thai Mental State Exam	POCD defined as decline of ≥1 SD on cognitive test. POCD in n=20/110 (18.2%).	Hypertension not defined. Prevalence of hypertension not reported.	None	RR 3.75 (1.10, 11.53)	14/22
Swaminathan *et al.* (2002) USA	625	282	71%	CABG	61 ± 10	6 weeks	4 factors of cognitive domains derived from 7 neuro-psychological tests	POCD defined as decline of ≥1SD on any of the 4 cognitive domains. POCD in n=112/282 (39.7%).	Hypertension not defined. Hypertension in n=173/282 (61.3%) of patients.	None	68/112 (60.7%)^c^ with POCD have hypertension. 105/170 (61.8%)^c^ without POCD have hypertension.	19/22
Kadoi & Goto (2006) Japan	95	88	80%	CABG General anesthesia	62 ± 11	6 months	5 neuro-psychological tests; MMSE	Definition of POCD unclear. POCD in n=24/88 (27.3%).	Hypertension not defined. Hypertension in n=49/88 (55.7%) of patients.	None	RR 1.5 (0.9, 1.8)	11/22
Bitsch *et al.* (2006) Denmark	100	96	29%	Hip fracture Regional and/or general anesthesia	POCD group: 86 (77 – 85) No POCD group: 81 (83 – 93)	7 days	MMSE “Major decline” defined as decline of ≥50% on MMSE.	“Major decline” used as POCD in present analysis. POCD in n=17/96 (17.7%). Note that for n=17/96 (17.7%), baseline assessment was in “early postoperative” phase.	Hypertension not defined. Hypertension in n=36/96 (37.5%) of patients.	None	7/17 (41.2%) with “major decline” have hypertension. 29/79 (36.7%) without “major decline” have hypertension.	20/22
Baba *et al.* (2007) Japan	218	218	70%	CABG General anesthesia	71 ± 6	7 days	4 neuro-psychological tests	POCD defined as decline of ≥20% on ≥ 3 tests. POCD in n=39/218 (17.9%).	Hypertension defined as “history of hypertension with anti-hypertensive medication”. Hypertension in n=170/218 (78.0%) of patients.	None.	30/39 (76.9%) with POCD have hypertension. 140/179 (78.2%) without POCD have hypertension.	16/22
Koch *et al.* (2007) USA	24	22	41%^b^	Knee/hip replace-ment surgery Spinal/general anesthesia	74 ± 6^b^	3 months	11 neuro-psychological tests	POCD defined as decline of ≥20% on ≥2 tests. POCD in n=10/22 (45.5%).	Hypertension not defined. Hypertension in n=14/22 (63.6%) of patients.	None	8/10 (80.0%) with POCD have hypertension. 6/12 (50.0%) without POCD have hypertension.	14/22
Mathew *et al.* (2007) USA	677	513	71%	CABG Anesthesia unreported	61 ± 10	6 weeks	4 factors of cognitive domains derived from 5 neuropsychological tests	POCD defined as ≥1 SD change on ≥1 of the 4 factor scores. POCD in n=152/443 (34.3%).	Hypertension not defined. Hypertension in n= 317/513 (61.8%) of patients.	None	113/183 (61.8%) with POCD have hypertension. 204/330 (61.8%) without POCD have hypertension.	19/22
Hong *et al.* (2008) South Korea	103	100	38%	Valvular heart surgery General anesthesia	53 ± 11	7 days	MMSE, TMT-A, Grooved Pegboard “Impairment” defined as: MMSE: decline ≥3 points; TMT-A/Grooved Pegboard: ≥20% increase in time	POCD defined as impairment on ≥1 of 3 tests. POCD in n=23/100 (23.0%).	Hypertension not defined. Hypertension in n=24/100 (24.0%) of patients.	None	1/23 (4.3%) with POCD have hypertension. 23/77 (29.9%) without POCD have hypertension	17/22
Wilson *et al.* (2008) USA	22^d^	21^d^	76%	Carotid end-arterectomy General anesthesia	69 ± 8	1 day	5 neuro-psychological tests. For each test, calculation of RCI^a^ RCI scores used to derive ‘total deficit score’ according to point system (score range 0-6 for each test). Total deficit score summed across tests. Control group n=20.	POCD defined as total deficit score ≥2 SD mean change in total deficit score of control group. POCD in n=6/21 (28.6%).	Hypertension defined as systolic blood pressure >140 mmHg or use of anti-hypertensive medication. Hypertension in n=17/21 (81.0%) of patients.	None	21/33 (63.6%) with POCD have hypertension^d^. 97/153 (63.4%) without POCD have hypertension^d^ (based on report on N=186)	16/22
Slater *et al.* (2009) USA	265	240	84%	CABG Anesthesia unreported	65 ± 10	3 months	5 neuro-psychological tests; MMSE	POCD defined as ≥1 SD decline on ≥1 tests. POCD in n=143/240 (59.6%).	Hypertension not defined. Hypertension in n=188/240 (78.3%) of patients.	None	116/143 (81.1%) with POCD have hypertension. 72/97 (74.2%) without POCD have hypertension.	20/22
Dieleman *et al.* (2009) Netherlands	281	240	73%	CABG Anesthesia unreported	61 ± 9	5 years	10 neuro-psychological tests. For each test, calculation of RCI^a^ and composite RCI. Control group n=112	POCD defined as composite RCI ≤ -1.96 and/or RCI ≤--1.96 in ≥2 tests, or diagnosis of dementia or stroke during follow-up. POCD in n=82/240 (34.2%).	Hypertension not defined. Hypertension in n=93/240 (38.8%) of patients.	None	23/82 (28.0%) with POCD have hypertension. 62/158 (39.2%) without POCD have hypertension. RR 1.04^c^ (*p*=0.89)	17/22
Norkiene *et al.* (2010) Lithuania	127	127	81%	CABG Anesthesia unreported	60 ± 7	7 to 9 days	6 neuro-psychological tests; MMSE	POCD defined as ≥1 SD decline on ≥2 tests. POCD in n=59/127 (46.5%).	Hypertension not defined. Hypertension in n=115/127 (90.6%) of patients	None	61/68 (89.7%) with POCD have hypertension. 54/59 (91.5%) without POCD have hypertension.	15/22
Kadoi *et al.* (2011a) Japan	129	124	80%	CABG General anesthesia	61 ± 5	7 days	5 neuro-psychological tests; MMSE	POCD defined as decline of ≥1 SD on ≥2 of 6 tests. POCD in n=30/124 (24.2%).	Hypertension not defined. Hypertension in n=90/124 (72.6%) of patients.	Age, carbon dioxide reactivity, jugular venous oxygen saturation, diabetic retinopathy, insulin therapy	RR 1.4 (1.0, 1.8)	13/22
Medi *et al.* (2013) Australia	120	120	72%	Radio- frequency ablation for atrial fibrillation General anesthesia	56 ± 10	3 months	8 neuro-psychological tests to calculate RCI^a^. RCI summed across tests and divided by SD of RCI sum of controls to obtain composite RCI. Control group n=30.	POCD defined as RCI <-1.96 on ≥2 tests and/or composite RCI <-1.96. POCD in n=15/120 (12.5%).	Hypertension not defined. Hypertension in n=49/120 (40.8%) of patients.	None	RR 0.5 (0.18, 1.6)	15/22
Plaschke *et al.* (2013) Germany	139	117	76%	CABG Anesthesia unreported	69 ± 8	3 months	6 neuro-psychological tests with 12 outcome variables used to calculate RCI^a^. RCI summed across tests and divided by SD of RCI sum of controls to obtain composite RCI. Control group n=34.	POCD defined as RCI ≥1.96 on ≥2 tests and/or composite RCI ≥1.96. POCD in n=30/117 (25.6%).	Hypertension not defined. Hypertension in n=116/117 (99.1%) of patients.	None	30/30 (100.0%) with POCD had hypertension. 86/87 (98.9%) without POCD had hypertension.	19/22
Xu *et al.* (2013) China	182	176	53%	Non-coronary bypass surgery (cardiac and non-cardiac surgery) General anesthesia	42 ± 19	3 to 5 days	MMSE. Calculation of RCI^a^. Control group n=16.	POCD defined as RCI≥1. POCD in n=58/176 (33.0%).	Hypertension not defined. Hypertension in n=25/176 (14.2%) of patients.	None	6/58 (10.3%) with POCD have hypertension. 19/118 (16.1%) without POCD have hypertension.	14/22
Fontes *et al.* (2013) USA	281	229	69%	CABG, valve or CABG + valve Anesthesia unreported	67 ± 10	1 year	4 factors of cognitive domains derived from 5 neuro-psychological tests. Mean of 4 factor scores used to derive “composite cognitive index score” (CCI). “Cognitive recovery” defined as CCI at 1 year ≥ CCI at baseline. Analysis sample included only patients who showed initial decline between baseline and 6-week follow-up.	“No cognitive recovery” used as POCD in present analysis. POCD in n=126/229 (55.0%).	Hypertension not defined. Hypertension in n=160/229 (69.9%) of patients.	None	69/103 (67.0%) with “cognitive recovery” have hypertension. 91/126 (72.2%) without “cognitive recovery” have hypertension.	18/22
Joudi *et al.* (2014) Iran	171	171	Unreported.	Off-pump CABG General anesthesia	64 ± 10	1 day	MMSE	Unclear definition of POCD. POCD in n=129/171 (75.4%).	Hypertension not defined. Hypertension in n=115/171 (67.3%) of patients.	None	80/129 (61.9%) with POCD have hypertension. 35/42 (83.7%) without POCD have hypertension.	13/22
Zhu *et al.* (2014) China	313	205	51%	Total hip replacement surgery Spinal or general anesthesia	75 ± 6	7 days	MMSE	POCD defined as ≥1 SD decline on MMSE. POCD in n=56 (27.3%).	Hypertension not defined. Hypertension in n=100/205 (48.8%) of patients.	None	29/56 (51.8%) with POCD have hypertension. 71/149 (47.7%) without POCD have hypertension.	15/22
Heyer *et al.* (2015) USA	662	585	65%	Carotid end- arterectomy General anesthesia	34.4% ≥75 years old	1 day	Unclear number of neuro-psychological tests of 4 cognitive domains. Calculation of RCI^a^. Control group n=156.	POCD defined as ≥2 SD worse performance on ≥2 cognitive domains and/or ≥1.5 SD worse performance on all 4 cognitive domains. POCD in n=145/585 (24.8%).	Hypertension not defined. Hypertension in n=338/585 (57.8%) of patients.	None	84/145 (57.9%) with POCD have hypertension. 254/440 (57.7%) without POCD have hypertension.	17/22
Shoair *et al.* (2015)^e^	69	69	33%	Noncardiac surgery. Regional and/or general anesthesia	71 ± 5	3 months	5 neuro-psychological tests Calculation of RCI^a^ RCI summed across tests and divided by SD of RCI sum of controls to obtain composite RCI. Control group n=54.	POCD defined RCI <1.96 on ≥2 tests and/or composite RCI <1.96. POCD in n=11/69 (15.9%)	Hypertension defined by combination of self-report and verification on basis of medical records. Hypertension in n=38/69 (55.1%) of patients.	None	5/11 (45.5%) with POCD have hypertension. 33/58 (56.9%) without POCD have hypertension.	19/22

All data refer to analysis sample that completed follow-up, unless otherwise indicated. CABG, coronary artery bypass grafting; CI, confidence interval; IQ, interquartile range; MMSE, Mini Mental State Examination; RCI, reliable change index; RR, relative risk; SD, standard deviation; STROBE, Strengthening the Reporting of Observational Studies in Epidemiology; TMT-A, Trail-Making Test A. ^a^formula for Reliable Change Index (RCI; often referred to as ‘z-score’ in original publication): RCI = (change score of patient group – change score of control group)/SD of change score of control group. ^b^based on total sample enrolled into study (data on analysis sample completing follow-up unreported). ^c^original reporting. Discrepancy with calculated RR for meta-analysis assumed due to unreported N missing (leading to total N in analyses being different to the ones reported in original article). ^d^total N uncertain on basis of article. ^e^unpublished data.

## References

[1] Evered L.A., Silbert B., Scott D.A. (2015). The impact of peri-operative period on cognition in older individuals.. J Pharm Practice Res.

[2] Rundshagen I. (2014). Postoperative cognitive dysfunction.. Dtsch. Arztebl. Int..

[3] Androsova G., Krause R., Winterer G., Schneider R. (2015). Biomarkers of postoperative delirium and cognitive dysfunction.. Front. Aging Neurosci..

[4] Ahlgren E., Lundqvist A., Nordlund A., Aren C., Rutberg H. (2003). Neurocognitive impairment and driving performance after coronary artery bypass surgery.. Eur. J. Cardiothorac. Surg..

[5] Funder K.S., Steinmetz J., Rasmussen L.S. (2009). Cognitive dysfunction after cardiovascular surgery.. Minerva Anestesiol..

[6] Plaschke K., Hauth S., Jansen C., Bruckner T., Schramm C., Karck M., Kopitz J. (2013). The influence of preoperative serum anticholinergic activity and other risk factors for the development of postoperative cognitive dysfunction after cardiac surgery.. J. Thorac. Cardiovasc. Surg..

[7] Gallo L.C., Malek M.J., Gilbertson A.D., Moore J.L. (2005). Perceived cognitive function and emotional distress following coronary artery bypass surgery.. J. Behav. Med..

[8] Monk T.G., Weldon B.C., Garvan C.W., Dede D.E., van der Aa M.T., Heilman K.M., Gravenstein J.S. (2008). Predictors of cognitive dysfunction after major noncardiac surgery.. Anesthesiology.

[9] Heyer E.J., Mergeche J.L., Wang S., Gaudet J.G., Connolly E.S. (2015). Impact of cognitive dysfunction on survival in patients with and without statin use following carotid endarterectomy.. Neurosurgery.

[10] Sarki AM, Nduka CU, Stranges S, Kandala NB, Uthman OA (2015). Prevalence of hypertension in low- and middle-income countries: A systematic review and meta-analysis.. Medicine.

[11] McDonald M., Hertz R.P., Unger A.N., Lustik M.B. (2009). Prevalence, awareness, and management of hypertension, dyslipidemia, and diabetes among United States adults aged 65 and older.. J. Gerontol. A Biol. Sci. Med. Sci..

[12] Schwartsmann C.R., Spinelli Lde.F., Boschin L.C., Yépez A.K., Crestani M.V., Silva M.F. (2015). Correlation between patient age at total hip replacement surgery and lifeexpectancy.. Acta Ortop. Bras..

[13] Preston S.D., Southall A.R., Nel M., Das S.K. (2008). Geriatric surgery is about disease, not age.. J. R. Soc. Med..

[14] Gillespie CD, Hurvitz KA (2013). Prevalence of hypertension and controlled hypertension - United States, 2007-2010.. MMWR supplements.

[15] Van den Berg E, Kloppenborg RP, Kessels RPC, Kappelle LJ, Biessels GJ (2009). Type 2 diabetes mellitus, hypertension, dyslipidemia and obesity: A systematic comparison of their impact on cognition.. Biochimicia et Biophysica Acta.

[16] Zaal I.J., Devlin J.W., Peelen L.M., Slooter A.J. (2015). A systematic review of risk factors for delirium in the ICU.. Crit. Care Med..

[17] Alberti K.G., Eckel R.H., Grundy S.M., Zimmet P.Z., Cleeman J.I., Donato K.A., Fruchart J.C., James W.P., Loria C.M., Smith S.C., International Diabetes Federation Task Force on Epidemiology and Prevention, Hational Heart, Lung, and Blood Institute, American Heart Association, World Heart Federation, International Atherosclerosis Society, International Association for the Study of Obesity (2009). Harmonizing the metabolic syndrome: A joint interim statement of the International Diabetes Federation Task Force on Epidemiology and Prevention; National Heart, Lung, and Blood Institute; American Heart Association; World Heart Federation; International Atherosclerosis Society; and International Association for the Study of Obesity.. Circulation.

[18] Feinkohl I., Winterer G., Pischon T. (2016). Obesity and post-operative cognitive dysfunction: a systematic review and meta-analysis.. Diabetes Metab. Res. Rev..

[19] Feinkohl I., Winterer G., Pischon T. (2017). Diabetes, glycemia and risk of post-operative cognitive dysfunction: A meta-analysis.. Diabetes Metab. Res. Rev..

[20] Escobar L., Escobar R., Cordero-Ampuero J. (2006). Previous medical problems in 326 consecutive hip fracture patients.. Hip Int.

[21] He J., Bazzano L.A. (2000). Effects of lifestyle modification on treatment and prevention of hypertension.. Curr. Opin. Nephrol. Hypertens..

[22] Stroup D.F., Berlin J.A., Morton S.C., Olkin I., Williamson G.D., Rennie D., Moher D., Becker B.J., Sipe T.A., Thacker S.B. (2000). Meta-analysis of observational studies in epidemiology: A proposal for reporting. Meta-analysis Of Observational Studies in Epidemiology (MOOSE) group.. JAMA.

[23] Moher D., Liberati A., Tetzlaff J., Altman D.G., PRISMA Group (2009). Preferred reporting items for systematic reviews and meta-analyses: The PRISMA statement.. PLoS Med..

[24] Davies H.T., Crombie I.K., Tavakoli M. (1998). When can odds ratios mislead?. BMJ.

[25] Shoair O.A., Grasso Ii M.P., Lahaye L.A., Daniel R., Biddle C.J., Slattum P.W. (2015). Incidence and risk factors for postoperative cognitive dysfunction in older adults undergoing major noncardiac surgery: A prospective study.. J. Anaesthesiol. Clin. Pharmacol..

[26] Kelly M.P., Garron D.C., Javid H. (1980). Carotid artery disease, carotid endarterectomy, and behavior.. Arch. Neurol..

[27] Di Carlo A., Perna A.M., Pantoni L., Basile A.M., Bonacchi M., Pracucci G., Trefoloni G., Bracco L., Sangiovanni V., Piccini C., Palmarini M.F., Carbonetto F., Biondi E., Sani G., Inzitari D. (2001). Clinically relevant cognitive impairment after cardiac surgery: A 6-month follow-up study.. J. Neurol. Sci..

[28] Bitsch M.S., Foss N.B., Kristensen B.B., Kehlet H. (2006). Acute cognitive dysfunction after hip fracture: Frequency and risk factors in an optimized, multimodal, rehabilitation program.. Acta Anaesthesiol. Scand..

[29] Suksompong S., Prakanratrana U., Chumpathong S., Sriyoschati S., Pornvilawan S. (2002). Neuropsychological alterations after coronary artery bypass graft surgery.. J. Med. Assoc. Thai..

[30] Kadoi Y., Goto F. (2006). Factors associated with postoperative cognitive dysfunction in patients undergoing cardiac surgery.. Surg. Today.

[31] Egger M., Davey Smith G., Schneider M., Minder C. (1997). Bias in meta-analysis detected by a simple, graphical test.. BMJ.

[32] Bo S., Gentile L., Cavallo-Perin P., Vineis P., Ghia V. (1999). Sex and BMI-related differences in risk factors for coronary artery disease in patients with type 2 diabetes mellitus.. Acta Diabetol..

[33] STROBE Initiative (2007). STROBE checklist for cohort studies, Version 4..

[34] Kadoi Y., Saito S., Fujita N., Goto F. (2005). Risk factors for cognitive dysfunction after coronary artery bypass graft surgery in patients with type 2 diabetes.. J. Thorac. Cardiovasc. Surg..

[35] Kadoi Y., Kawauchi C., Ide M., Kuroda M., Takahashi K., Saito S., Fujita N., Mizutani A. (2011). Preoperative depression is a risk factor for postoperative short-term and long-term cognitive dysfunction in patients with diabetes mellitus.. J. Anesth..

[36] Heyer E.J., Mergeche J.L., Anastasian Z.H., Kim M., Mallon K.A., Connolly E.S. (2014). Arterial blood pressure management during carotid endarterectomy and early cognitive dysfunction.. Neurosurgery.

[37] Kadoi Y., Kawauchi C., Kuroda M., Takahashi K., Saito S., Fujita N., Mizutani A. (2011). Association between cerebrovascular carbon dioxide reactivity and postoperative short-term and long-term cognitive dysfunction in patients with diabetes mellitus.. J. Anesth..

[38] Kazui H., Mori E., Ohkawa S., Okada T., Kondo T., Sakakibara R., Ueki O., Nishio Y., Ishii K., Kawaguchi T., Ishikawa M., Takeda M. (2013). Predictors of the disappearance of triad symptoms in patients with idiopathic normal pressure hydrocephalus after shunt surgery.. J. Neurol. Sci..

[39] Smith M.H., Wagenknecht L.E., Legault C., Goff D.C., Stump D.A., Troost B.T., Rogers A.T. (2000). Age and other risk factors for neuropsychologic decline in patients undergoing coronary artery bypass graft surgery.. J. Cardiothorac. Vasc. Anesth..

[40] Swaminathan M., McCreath B.J., Phillips-Bute B.G., Newman M.F., Mathew J.P., Smith P.K., Blumenthal J.A., Stafford-Smith M., Perioperative Outcomes Research Group (2002). Serum creatinine patterns in coronary bypass surgery patients with and without postoperative cognitive dysfunction.. Anesth. Analg..

[41] Wilson D.A., Mocco J., DAmbrosio A.L., Komotar R.J., Zurica J., Kellner C.P., Hahn D.K., Connolly E.S., Liu X., Imielinska C., Heyer E.J. (2008). Post-carotid endarterectomy neurocognitive decline is associated with cerebral blood flow asymmetry on post-operative magnetic resonance perfusion brain scans.. Neurol. Res..

[42] Koch S., Forteza A., Lavernia C., Romano J.G., Campo-Bustillo I., Campo N., Gold S. (2007). Cerebral fat microembolism and cognitive decline after hip and knee replacement.. Stroke.

[43] Mathew J.P., Podgoreanu M.V., Grocott H.P., White W.D., Morris R.W., Stafford-Smith M., Mackensen G.B., Rinder C.S., Blumenthal J.A., Schwinn D.A., Newman M.F., PEGASUS Investigative Team (2007). Genetic variants in P-selectin and C-reactive protein influence susceptibility to cognitive decline after cardiac surgery.. J. Am. Coll. Cardiol..

[44] Hong S.W., Shim J.K., Choi Y.S., Kim D.H., Chang B.C., Kwak Y.L. (2008). Prediction of cognitive dysfunction and patients outcome following valvular heart surgery and the role of cerebral oximetry.. Eur. J. Cardiothorac. Surg..

[45] Slater J.P., Guarino T., Stack J., Vinod K., Bustami R.T., Brown J.M., Rodriguez A.L., Magovern C.J., Zaubler T., Freundlich K., Parr G.V. (2009). Cerebral oxygen desaturation predicts cognitive decline and longer hospital stay after cardiac surgery.. Ann. Thorac. Surg..

[46] Dieleman J., Sauër A-M., Klijn C., Nathoe H., Moons K., Kalkman C., Kappelle J., Van Dijk D. (2009). Presence of coronary collaterals is associated with a decreased incidence of cognitive decline after coronary artery bypass surgery.. Eur. J. Cardiothorac. Surg..

[47] Norkienė I., Samalavičius R., Misiūrienė I., Paulauskienė K., Budrys V., Ivaškevičius J. (2010). Incidence and risk factors for early postoperative cognitive decline after coronary artery bypass grafting.. Medicina (Kaunas).

[48] Medi C., Evered L., Silbert B., Teh A., Halloran K., Morton J., Kistler P., Kalman J. (2013). Subtle post-procedural cognitive dysfunction after atrial fibrillation ablation.. J. Am. Coll. Cardiol..

[49] Baba T., Goto T., Maekawa K., Ito A., Yoshitake A., Koshiji T. (2007). Early neuropsychological dysfunction in elderly high-risk patients after on-pump and off-pump coronary bypass surgery.. J. Anesth..

[50] Xu T., Bo L., Wang J., Zhao Z., Xu Z., Deng X., Zhu W. (2013). Risk factors for early postoperative cognitive dysfunction after non-coronary bypass surgery in Chinese population.. J. Cardiothorac. Surg..

[51] Fontes M.T., Swift R.C., Phillips-Bute B., Podgoreanu M.V., Stafford-Smith M., Newman M.F., Mathew J.P., Neurologic Outcome Research Group of the Duke Heart Center (2013). Predictors of cognitive recovery after cardiac surgery.. Anesth. Analg..

[52] Joudi M., Fathi M., Harati H., Joudi M., Izanloo A., Rahdari A., Soltani G. (2014). Evaluating the incidence of cognitive disorder following off-pump coronary artery bypasses surgery and its predisposing factors.. Anesth. Pain Med..

[53] Zhu S-H., Ji M-H., Gao D-P., Li W-Y., Yang J-J. (2014). Association between perioperative blood transfusion and early postoperative cognitive dysfunction in aged patients following total hip replacement surgery.. Ups. J. Med. Sci..

[54] Stroobant N., van Nooten G., De Bacquer D., Van Belleghem Y., Vingerhoets G. (2008). Neuropsychological functioning 35 years after coronary artery bypass grafting: does the pump make a difference?. Eur. J. Cardiothorac. Surg..

[55] An J., Fang Q., Huang C., Qian X., Fan T., Lin Y., Guo Q. (2011). Deeper total intravenous anesthesia reduced the incidence of early postoperative cognitive dysfunction after microvascular decompression for facial spasm.. J. Neurosurg. Anesthesiol..

[56] Khan A.H., Khilji S.A. (2005). Neurological outcome after coronary artery bypass surgery.. J. Ayub Med. Coll. Abbottabad.

[57] Kotekar N., Kuruvilla C.S., Murthy V. (2014). Post-operative cognitive dysfunction in the elderly: A prospective clinical study.. Indian J. Anaesth..

[58] Kilo J., Czerny M., Gorlitzer M., Zimpfer D., Baumer H., Wolner E., Grimm M. (2001). Cardiopulmonary bypass affects cognitive brain function after coronary artery bypass grafting.. Ann. Thorac. Surg..

[59] Chen C.W., Lin C.C., Chen K.B., Kuo Y.C., Li C.Y., Chung C.J. (2014). Increased risk of dementia in people with previous exposure to general anesthesia: A nationwide population-based case-control study.. Alzheimers Dement..

[60] Yu WK, Chen YT, Wang SJ, Kuo SC, Shia BC, Liu CJ (2015). Cataract surgery is associated with a reduced risk of dementia: A nationwide population-based cohort study.. Eur J Neurol.

[61] Yocum G.T., Gaudet J.G., Teverbaugh L.A., Quest D.O., McCormick P.C., Connolly E.S., Heyer E.J. (2009). Neurocognitive performance in hypertensive patients after spine surgery.. Anesthesiology.

[62] Pereira-Filho A.A., Pereira A.G., Pereira-Filho N.A., Lima L.C., da Costa J.C., Kraemer J.L., Portuguez M.W. (2014). Long-term behavioral and cognitive outcomes following clipping for incidental unruptured intracranial aneurysms.. Neuropsychology.

[63] Tully P.J., Baker R.A., Knight J.L., Turnbull D.A., Winefield H.R. (2009). Neuropsychological function 5 years after cardiac surgery and the effect of psychological distress.. Arch. Clin. Neuropsychol..

[64] Wolman R.L., Nussmeier N.A., Aggarwal A., Kanchuger M.S., Roach G.W., Newman M.F., Mangano C.M., Marschall K.E., Ley C., Boisvert D.M., Ozanne G.M., Herskowitz A., Graham S.H., Mangano D.T. (1999). Cerebral injury after cardiac surgery: identification of a group at extraordinary risk. Multicenter Study of Perioperative Ischemia Research Group (McSPI) and the Ischemia Research Education Foundation (IREF) Investigators.. Stroke.

[65] Tully P.J., Hanon O., Cosh S., Tzourio C. (2016). Diuretic antihypertensive drugs and incident dementia risk: A systematic review, meta-analysis and meta-regression of prospective studies.. J. Hypertens..

[66] McGuinness B, Todd S, Passmore P, Bullock R (2009). Blood pressure lowering in patients without prior cerebrovascular disease for prevention of cognitive impairment and dementia.. Cochrane Database Syst Rev.

[67] Sharp S.I., Aarsland D., Day S., Sønnesyn H., Ballard C., Alzheimers Society Vascular Dementia Systematic Review Group (2011). Hypertension is a potential risk factor for vascular dementia: Systematic review.. Int. J. Geriatr. Psychiatry.

[68] Craft S. (2009). The role of metabolic disorders in Alzheimer disease and vascular dementia: Two roads converged.. Arch. Neurol..

[69] Dubow J., Fink M.E. (2011). Impact of hypertension on stroke.. Curr. Atheroscler. Rep..

[70] PROGRESS Collaborative Group (2001). Randomised trial of a perindopril-based blood-pressure-lowering regimen among 6,105 individuals with previous stroke or transient ischaemic attack.. Lancet.

[71] Benarroch E.E. (2007). Neurovascular unit dysfunction: A vascular component of Alzheimer disease?. Neurology.

[72] Perrotta M., Lembo G., Carnevale D. (2016). Hypertension and dementia: Epidemiological and experimental evidence revealing a detrimental relationship.. Int. J. Mol. Sci..

[73] Evered L., Silbert B., Scott D.A., Ames D., Maruff P., Blennow K. (2016). Cerebrospinal fluid biomarker for Alzheiumer disease predicts postoperative cognitive dysfunction.. Anesthesiology.

[74] Feinkohl I., Keller M., Robertson C.M., Morling J.R., McLachlan S., Frier B.M., Deary I.J., Strachan M.W., Price J.F. (2015). Cardiovascular risk factors and cognitive decline in older people with type 2 diabetes.. Diabetologia.

[75] McGuinness B, Craig D, Bullock R, Passmore P (2009). Statins for the prevention of dementia (review).. Cochrane Libr.

[76] Franklin S.S., Thijs L., Hansen T.W., OBrien E., Staessen J.A. (2013). White-coat hypertension: New insights from recent studies.. Hypertension.

[77] Gonçalves K.K., Silva J.I., Gomes E.T., Pinheiro L.L., Figueiredo T.R., Bezerra S.M. (2016). Anxiety in the preoperative period of heart surgery.. Rev. Bras. Enferm..

[78] Duron E., Hanon O. (2008). Hypertension, cognitive decline and dementia.. Arch. Cardiovasc. Dis..

[79] Cook D.J., Huston J., Trenerry M.R., Brown R.D., Zehr K.J., Sundt T.M. (2007). Postcardiac surgical cognitive impairment in the aged using diffusion-weighted magnetic resonance imaging.. Ann. Thorac. Surg..

[80] Hogue C.W., Murphy S.F., Schechtman K.B., Dávila-Román V.G. (1999). Risk factors for early or delayed stroke after cardiac surgery.. Circulation.

[81] Hoeymans N., Smit H.A., Verkleij H., Kromhout D. (1996). Cardiovascular risk factors in relation to educational level in 36 000 men and women in The Netherlands.. Eur. Heart J..

[82] Feinkohl I, Winterer G, Spies CD, Pischon T (2017). Cognitive reserve and the risk of postoperative cognitive dysfunction - A systematic review and meta-analysis.. Dtsch. Arztebl. Int..

[83] Odden M.C., Beilby P.R., Peralta C.A. (2015). Blood pressure in older adults: the importance of frailty.. Curr. Hypertens. Rep..

[84] Kojima G., Taniguchi Y., Iliffe S., Walters K. (2016). Frailty as a predictor of Alzheimer disease, vascular dementia, and all dementia among community-dwelling older people: A systematic review and meta-analysis.. J. Am. Med. Dir. Assoc..

